# Analysis of Geographic and Environmental Factors and Their Association with Cutaneous Melanoma Incidence in Canada

**DOI:** 10.1159/000524949

**Published:** 2022-06-09

**Authors:** Melissa Berman-Rosa, James Logan, Feras M. Ghazawi, Michelle Le, Santina Conte, Elena Netchiporouk, Ilya M. Mukovozov, Janelle Cyr, Ahmed Mourad, Wilson H. Miller, Joël Claveau, Thomas G. Salopek, Robert Gniadecki, Denis Sasseville, Elham Rahme, François Lagacé, Ivan V. Litvinov

**Affiliations:** ^a^Department of Family Medicine, McGill University, Montreal, Québec, Canada; ^b^Independent Consultant, MGIS, Ottawa, Ontario, Canada; ^c^Division of Dermatology, University of Ottawa, Ottawa, Ontario, Canada; ^d^Division of Dermatology, McGill University, Montreal, Québec, Canada; ^e^Department of Dermatology and Skin Sciences, University of British Columbia, Vancouver, British Columbia, Canada; ^f^Division of Dermatology, University of Toronto, Toronto, Ontario, Canada; ^g^Division of Dermatology, University of Calgary, Calgary, Alberta, Canada; ^h^Department of Medicine and Oncology, McGill University, Montreal, Québec, Canada; ^i^Division of Dermatology, Laval University, Quebec City, Québec, Canada; ^j^Division of Dermatology, University of Alberta, Edmonton, Alberta, Canada; ^k^Division of Clinical Epidemiology, McGill University, Montreal, Québec, Canada

**Keywords:** Melanoma, Cutaneous melanoma, Incidence, Risk factors, Canadian Urban Environmental Health Research Consortium, Canadian Cancer Registry, Climate, Geography, Heat, Rain, Temperature, Ultraviolet radiation, Ultraviolet index, Normalized difference vegetation index, Latitude

## Abstract

**Background:**

Over 90% of skin cancers including cutaneous melanoma (CM) are related directly to sun exposure. Despite extensive knowledge on ultraviolet radiation's (UVR) detrimental impact, many still fail to implement sun protection/sun avoidance. Human behavior, attitudes, and cultural norms of individuals and communities heavily depend on the surrounding climate/environment. In many instances, the climate shapes the culture/norms of the society. Canada has vast geographic/environmental differences.

**Methods:**

In the current ecological study, we sought to examine the relationship between various geographic and environmental factors and the distribution of CM incidence by Forward Sortation Area (FSA) postal code across Canada. CM incidence data were extracted from the Canadian Cancer Registry, while environmental data were extracted from the Canadian Urban Environmental Health Research Consortium (greenspace, as measured by the normalized difference vegetation index; annual highest temperature; absolute number and average length of yearly heat events; annual total precipitation [rain and snow]; absolute number and average length of events with precipitation [rain and snow]; and summer UVR index). The above geographic/environmental data by FSA were correlated with the respective CM incidence employing negative binomial regression model.

**Results:**

Our analysis highlights that increases in annual average temperature, summer UVR, and greenspace were associated with higher expected incidence of CM cases, while higher number of annual heat events together with highest annual temperature and higher average number of annual rain events were associated with a decrease in CM incidence rate. This study also highlights regional variation in environmental CM risk factors in Canada.

**Conclusions:**

This national population-based study presents clinically relevant conclusions on weather/geographic variations associated with CM incidence in Canada and will help refine targeted CM prevention campaigns by understanding unique weather/geographic variations in high-risk regions.

## Introduction

Cutaneous melanoma (CM) causes more deaths than any other skin cancer [1], accounting for ∼1.9% and 1.2% of all cancer deaths in males and females, respectively, in Canada [2]. Globally, in 2015, >350,000 individuals were affected by CM [3]. The regions with the highest age-standardized incidence rates (ASIR) per 100,000 individuals include Australia (54.1), North America (21.0), Western Europe (15.6), Central Europe (8.3), and Eastern Europe (7.8) [4]. In Canada (1992–2010 ASIR of 9.6), CM is the 8th most commonly diagnosed malignancy according to the Canadian Cancer Society.

For decades, the link between ultraviolet (UV) radiation exposure and the risk of developing skin cancer has been well-established. Solar and artificial UV exposure plays a critical role in the development of: (a) melanoma, keratinocyte carcinoma, Merkel cell carcinoma; (b) skin photoaging; and (c) direct or indirect causation of other skin diseases (e.g., polymorphous light eruption, solar urticaria, chronic actinic dermatitis, melasma, lupus erythematosus, and rosacea) [5, 6, 7]. UV/solar radiation, combined with many host factors and other determinants (e.g., Fitzpatrick skin phototype, number of melanocytic nevi, personal/family history of melanoma and other skin cancers, history of sunburns, therapy with psoralen UVA or broadband UVB, immunosuppression, and genetic factors/mutations) determine the individual's ultimate risk for this deadly disease [8, 9]. Despite extensive knowledge on UV radiation's (UVR) detrimental impact, many people still fail to implement sun protection/sun avoidance (e.g., cumulative sun exposure, sunburns, tanning bed use), probably due to the numerous beliefs around sun exposure (e.g., requirement of excessive sun exposure to produce endogenous vitamin D, cultural appeal of a skin tan as being “healthy,” attractive, and a sign of affluence) [10, 11].

We detailed in previous studies the data on Canadian CM epidemiologic trends and documented the burden of this cancer (crude and age-standardized rates) for the period of 1992–2010 [12, 13, 14]. Our results showed that the maritime provinces of Nova Scotia (crude incidence rate of 18.87 cases per 100,000 individuals per year) and Prince Edward Island (18.82) had notably high CM incidence rates. CM incidence rates in New Brunswick (15.02), British Columbia (15.41), and Ontario (14.19) were higher but comparable to the national average of 12.29 cases per 100,000 individuals per year. CM incidence in the Prairie Provinces of Manitoba (10.95), Saskatchewan (11.62), and Alberta (11.88) was just below the national average. Quebec had a lower incidence rate (7.06), but the methodology used to calculate melanoma incidence differed significantly in Quebec (managed by Le Registre Québécois du Cancer) compared to the rest of Canada (managed by the Canadian Cancer Registry [CCR]). Also, the Northern Territories (4.69) had a significantly lower rate, as expected, due to their geographic location away from the equator (i.e., higher latitude) leading to less intense UV index for most of the year. The overall darker skin phototype of First Nations communities, accounting for the majority of the population in these regions [14], combined with a colder climate, plausibly decreases the likelihood of sun exposure and UV skin damage. Notably, detailed analysis of incidence by first three entries of a postal code (also known as the Forward Sortation Area [FSA]) highlighted the regions of Nova Scotia and Prince Edwards Island, Southern Ontario/British Columbia, and coastal New Brunswick as having significantly higher rates than Northern Ontario/British Columbia, Newfoundland and Labrador, and the Prairie Provinces. We also documented an inverse relationship between CM incidence and geographic latitude in Canada [14].

In the current ecological study, we sought to examine the relationship between various geographic and environmental factors and the distribution of melanoma incidence across Canada based on our previously analyzed 1992–2010 data [12, 14]. In order to elucidate potential contributors to the ever-increasing CM trend and to better understand their possible impact on human behavior and community norms in relation to sun exposure practices, we explored the potential existence of region-level patterns of associations between weather events (rain, snow, heat), presence of greenspace, as defined by the normalized difference vegetation index (NDVI), UVR index, and the melanoma incidence across Canada, a large and geographically diverse region of the world.

## Methods

**Fig. 1:**
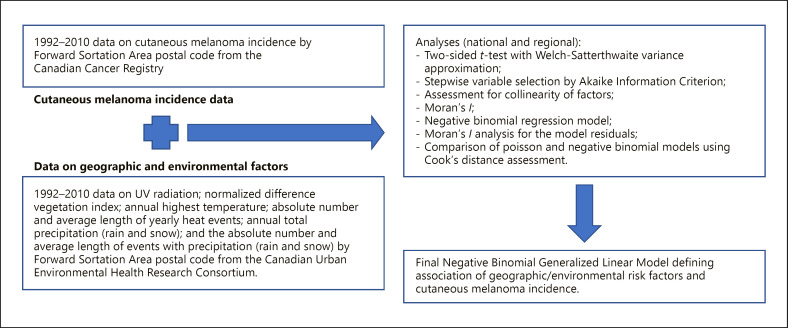
Flowchart of databases and methods.

Flowchart of methods is presented in Figure 1.

### Melanoma Incidence

This study was conducted in accordance with the QICSS-RDC-668035 and 13-SSH-MCG-3749-S001 protocols approved by the Social Sciences and Humanities Research Council of Canada and the Québec Inter-University Centre for Social Statistics, respectively. Data on the incidence of CM for the period 1992–2010 were obtained from the CCR. Canada's territories were excluded due to low number of melanoma cases throughout the time period. CM diagnosis was classified according to the International Classification of Diseases for Oncology (ICD-O)-3, ICD-9, and ICD-10 codes for all subtypes of CM, in a similar manner as previously reported [13, 14, 15, 16, 17, 18]. The CCR is a dynamic database of Canadian residents across the country's 12 provinces and territories (excluding Québec) who have been diagnosed with primary tumors from 1992 to 2017, whether alive or dead. Data for residents of Quebec are contained in the Le Registre Québécois du Cancer database, which, unfortunately, assesses/counts CM cases differently than the CCR. For this study, only the CCR data were used. Further details concerning these databases have been described at length in our previous publications [12, 14]. Incidence analyses were carried out at the levels of FSAs. In Canada, postal codes consist of letters and numbers (e.g., H3G 1A4), where the first 3 entries (e.g., H3G) are a set of well-defined and stable geographical areas in the country, with more than 1,600 FSAs across the country [19]. Population counts for FSAs were obtained from Statistics Canada Census of Population for the years 1996, 2001, 2006, and 2011.

### Environmental Data

The Canadian Urban Environmental Health Research Consortium (CANUE) is funded under the Canadian Institutes of Health Research (CIHR), Environments and Health Signature Initiative and facilitates the linkage of extensive geospatial exposure data to the wealth of established cohorts and administrative health data holdings [20]. For the period of 1992–2010, we retrieved the data on the following environmental factors: UVR [21]; greenspace, as measured by the NDVI [22, 23, 24, 25]; annual highest temperature; absolute number and average length of yearly heat events; annual total precipitation (rain and snow); and the absolute number and average length of events with precipitation (rain and snow).

### Procedure and Statistical Analysis

Incidence rates of CM across Canada for the 1992–2010 period were obtained from our previously published population-based analyses [12, 14] and are presented here as ASIR per 100,000 individuals per year. Previous CM incidence mapping highlighted 8 regions within Canada where melanoma overall was higher or average/lower than the national average [14]. Hence, FSAs were divided into 8 different geographic regions based on low versus high CM incidence (online suppl. Table S1) (for all online suppl. material, see www.karger.com/doi/10.1159/000524949); high incidence regions: (1) maritime provinces of Nova Scotia, Prince Edward Island and New Brunswick; (2) Southern Ontario; (3) southern British Columbia and low incidence regions: (4) Northern Ontario; (5) Newfoundland and Labrador; (6) Saskatchewan and Manitoba; (7) Alberta; (8) northern British Columbia. Each FSA within a given region was subsequently categorized as high- or low-risk based on whether the melanoma incidence was statistically higher than the Canadian-average (high-risk group), statistically lower than, or no different than the national incidence rate (low-risk group). An independent two-sided *t* test with Welch-Satterthwaite variance approximation that does not assume equal variances in the two groups was used to compare the high-risk FSAs to the low-risk FSAs country-wide, based on the above highlighted environmental characteristics.

Means, standard deviations, and interquartile ranges were calculated to summarize the measurement of each risk factor over the 19 years of the study period. Linear associations and correlation coefficients were assessed for each pair of independent variables. Selection between collinear independent variables was determined by pairwise comparison of *p* values from univariate negative binomial regression analyses. A stepwise variable selection by Akaike Information Criterion was performed with an offset term adjusting for average FSA population during the study period. Average of data points for 1992–2010 years was used. Overall and individual variance inflation factors for the resulting model were generated by the mctest package to assess for collinearity (https://cran.r-project.org/web/packages/mctest/mctest.pdf) (e.g., mean annual rain days and annual average temperature).

### Moran's *I*

To identify whether regional geographic effects might drive high CM rates independent of the modeled risk factors, we assessed the data for any influence of spatial autocorrelation. Moran's *I* was calculated using Environmental Systems Research Institute ArcGIS Pro software (https://www.esri.com/en-us/arcgis/products/arcgis-pro/overview) to measure spatial clustering of ASIR, an indication of the presence or absence of groups of adjacent FSAs that exhibit unusually high incidence compared to other neighboring FSAs.

The Moran's *I* statistic is measured on a scale of 1 (similar rates tend to be geographically clustered) to −1 (completely uniform pattern); a result of 0 represents complete spatial randomness [26]. As a supplementary strategy to assess any existence of clustered patterns, incidence rates were also mapped according to the Anselin Local Moran's *I* [27], which identifies whether each feature (in this case, each FSA) exhibits similarly high or low rates. As the Local Moran's *I* analysis did not detect high-incidence FSA clusters that departed from the high and low regional patterns described above (online suppl. Table S1), we chose not to include the multi-level effects for regions in the regression analyses.

### Negative Binomial Regression

We used the negative binomial regression model available in R (version 4.0.3) to analyze patterns of CM cases aggregated by Canadian FSAs. Notably, as the dependent variables (incidence of CM) show significant skewness to the right; are noncontinuous in nature; and cannot exhibit negative values, standard linear regression approaches are not well-suited. We used the negative binomial regression model available in R (version 4.0.3) to analyze patterns of CM cases aggregated by Canadian FSAs. These models are suitable for the analyses of count data that are skewed and where significant overdispersion may be a concern [28].

### Moran's *I* Analysis for the Model Residuals

Following stepwise model selection, Moran's *I* was calculated for model residuals to detect whether any spatial patterns remained in the model. The value of this analysis was compared to the value calculated in evaluation FSA clustering of age-standardized incidence. If unchanged from the autocorrelation statistic for FSA-level incidence, the value of the residual-based *I* statistic should suggest the presence of any remaining regional effects [29]. Comparison with the Moran's *I* value for FSA-level incidence was assessed as a measure of spatial structure in the data, after adjusting for environmental factors included in the final model.

## Results

Various types of geographic/environmental data for FSAs from high versus low CM incidence regions (online suppl. Table S1) were correlated with their respective melanoma incidence between 1992 and 2010. The value of Moran's *I* (0.16; *p* < 0.01) suggested a small amount of clustering was present among FSAs across the entirety of the tested regions (online suppl. Table S1). As aggregation of environmental variables to the level of FSA analysis units may account for geographic variance, a regionally clustered approach was determined as being suitable. This is supported by the Moran's *I* statistic calculated for the residuals in the model (0.086; *p* < 0.01). The measurement of spatial autocorrelation was roughly halved in the model. Hence, their spatial effects of high incidence FSA clusters might occur that extend beyond the geographic variation already controlled for by the modeled environmental variables.

Nationwide, significant differences in weather events were observed between CM high-incidence and low-incidence FSAs. Notably, high-incidence FSAs had higher annual average temperature (7.75°C vs. 6.31°C, *p* < 0.05), higher summer UVR (6,291 J/m^2^ vs. 6,108 J/m^2^, *p* < 0.05), and more surrounding green-space/vegetation, as measured by the NDVI (index) at a 1,000 m buffer zone (Table 1).

For regional comparisons, we evaluated high-risk FSAs in the maritime provinces (Nova Scotia, New Brunswick, and Prince Edward Island), Southern Ontario, and southern British Columbia areas versus the low-risk FSAs in adjacent regions (Table 2) via a two-tailed *t* test with Welch-Satterthwaite variance approximation, and significance was determined (alpha = 0.05). High-risk FSAs differed from the low-risk FSAs in that they had higher annual average temperature, higher summer UVR, and higher NDVI.

We modelled the expected counts of melanoma cases per FSA, as predicted by the variables identified in our nationwide comparison. Each variable was centered and scaled by the standard deviation (SD) and converted into *Z*-scores. Stepwise model selection resulted in two potential models that were comparable. In the first model, collinearity was detected between the average number of days of rain and annual average temperature. Thus, we focused on a model (Table 3) where average number of days of rain was excluded. In this final model, increase in annual average temperature, summer UVR, and greenspace as defined by the NDVI were associated with higher expected incidence of CM cases, while higher number of annual heat events together with highest annual temperature and higher average number of annual rain events were associated with a decrease in CM incidence rate.

Specifically, an increase of one SD (2.5°C) in the annual average temperature was associated with an increase in the number of expected CM incidence by 21.1% in a given region (IRR = 1.211, 95% CI: 1.155, 1.270). UVR increase in one SD (443.3 J/m^2^) or NDVI index increase by one SD (0.095) was associated with an increase in CM incidence by 13.7% (IRR: 1.137, 95% CI: 1.094, 1.182) and 16.7% (IRR = 1.167, 95% CI: 1.132, 1.202), respectively (Table 3). In contrast, increasing the annual average number of rain events in a region/FSA by one SD (7.6 events) was associated with a decrease in CM incidence in that area by ∼12% (IRR = 0.883, 95% CI: 0.846, 0.921). Increases in the annual number of heat events (1 heat event) or the highest annual temperature (SD: 1.57°C) were associated with decreasing CM incidence by 9.4% (IRR = 0.906, 95% CI: 0.831, 0.988) and 6.2% (IRR = 0.938, 95% CI: 0.904, 0.973), respectively. However, while significant (*p* < 0.05), the 95% confidence interval in the latter measurements approached 1, suggesting that these effects may be less important to the overall impact.

**Fig. 2:**
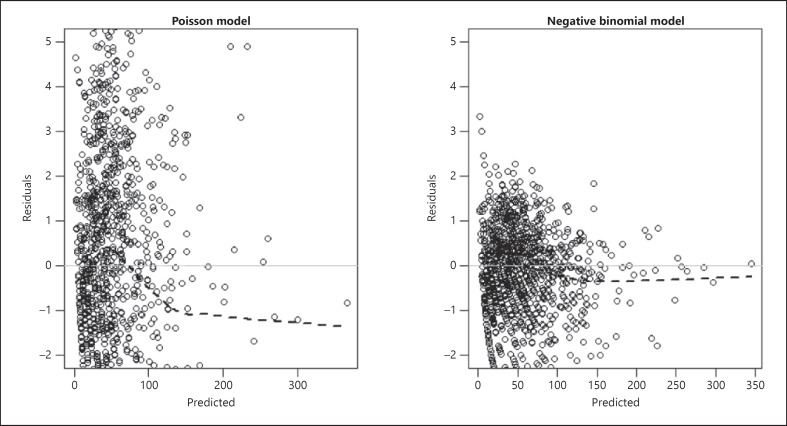
Plots of residuals versus predicted counts of melanoma cases for Poisson and negative binomial versions of the final model.

**Fig. 3:**
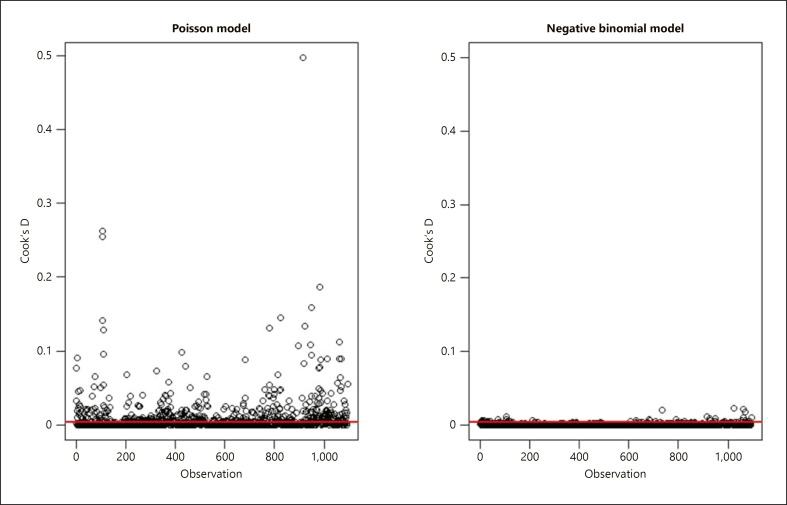
Cook's distance assessment of influential observations for the final model, contrasting Poisson and negative binomial distributions. Greater Cook's distances from the solid line indicate observations with greater influence on the model.

To evaluate whether the negative binomial distribution was more appropriate for the data analysis than the Poisson distribution, we used both to fit the variables in the final model and compared diagnostic plots (Fig. 2, 3). We plotted model residuals (the difference between the model-predicted count and the real number observed for each data point) against the values predicted by the model. The residual plot from the negative binomial regression model is displayed in Figure 2. The grey zero-line represents the line of perfect fit for the model. In other words, if every point was localized to this line, the model would have predicted every observation from the data. The further from this line the values fall, the less accurate the prediction is for that particular data point. The dotted line represents an approximation of how well the model performed at achieving this fit; this estimate for the negative binomial version of the final model was significantly closer to following the horizontal and minimizing residuals for most points.

The values of Cook's D for each observation show how much the model changes if that value is removed, serving as the visual summary of how much influence each data point has on the model (Fig. 3). Observations in the Poisson version of the model have much greater influence on the fitted model, whereas removal of any one observation, when using the negative binomial distribution, had very little effect on the remaining predicted values. As such, both sets of plots confirmed that the negative binomial model was the most appropriate (Table 3).

## Discussion

Our study for the first time evaluated numerous environmental parameters/potential environmental risk factors analyzed by Environment Canada (CANUE database) by FSA postal code and compared them to CM incidence in order to propose a model that accounts for the impact of environment/climate on the incidence of this cancer. Our data indicate that higher general annual temperature (i.e., more pleasant ambient temperature), availability of green spaces, and the UVR index are the three main factors associated with CM risk in Canada. This association may perhaps be explained by the impact of these factors on individual behavior and community norms [30, 31], leading to higher or lower rates of sun exposure, which in turn impacts CM incidence. On the other hand, higher number of rain and heat events together with the highest annual average temperature observed may tempt the public to stay indoors in air-conditioned facilities or prompt individuals to apply more clothing (to protect from the rain) [32], which is associated with lower CM incidence rates.

In support of our findings, studies in Australia have shown that with rising temperatures, adults are more likely to spend time outdoors, less likely to wear protective clothing, and more likely to get a sunburn [33]. It was observed that the likelihood of a sunburn approximately doubled when the temperature was 19–27°C compared to temperatures of ≤18°C [34, 35]. In contrast, one report established that at temperatures >27°C, the risk of sunburn decreased, as people seek shade for comfort reasons [34].

Human behavior, attitudes, and cultural norms of the community heavily depend on the surrounding climate/environment. In many instances, the climate shapes the culture/norms of the society. Inherently, geographic location and seasonal/weather variations have been linked to CM presumably by modulating the amount of UVR reaching the earth's surface [36]. Because over 90% of skin cancers are related directly to sun exposure, skin cancer can be prevented by using appropriate sun protection. The WHO INTERSUN program (https://www.who.int/initiatives/intersun-programme), created for the purpose of reducing the morbidity and mortality associated with skin cancer worldwide, recommends modifications of the physical environment for the provision of shade in public places [37]. Furthermore, the Ottawa Charter for Health Promotion outlines five action areas that require attention when population health-related issues are at stake, namely: (1) building healthy public policy (e.g., laws that improve health outcomes); (2) creating supportive environments (e.g., culture-sensitive initiatives); (3) strengthening community action (e.g., empowering community members); (4) developing personal skills (e.g., self-management); and (5) reorienting health services (i.e., moving from treatment- to health promotion-oriented service) [38]. Successful sun protection/melanoma awareness campaigns will have to strive to promote individual and social change, whereby changing community norms is central to the process. Hence, a detailed understanding of the climate/environment's role in high-risk CM regions is critical to the success of these campaigns.

Several targeted programs to help decrease UV radiation exposure have been conducted in different parts of the world. For instance, in Australia, the multi-component sustained Slip! Slop! Slap! and SunSmart (since 1980) campaigns [39, 40, 41, 42, 43, 44, 45, 46, 47, 48, 49, 50, 51] are credited as having an impact on individual behavior as well as broader societal changes, which led to the implementation of policies for hat-wearing and shade provision in childcare centers, primary schools, and workplaces; the increased availability/affordability of more effective sunscreens that extend protection time and filter a greater range of UV rays; the inclusion of sun protection items as a tax-deductible expense for outdoor workers; increased demand and availability of long-sleeved sun-protective swimwear; a ban on use of tanning beds in 2014; the provision of UV forecasts in weather reports; and in recent years a comprehensive program of grants for the provision of community shade [40, 48, 50, 52]. Even the fashion industry (e.g., the promotion of neck-to-knee Lycra swimwear for children) and building design to incorporate more shade-producing structures have been affected by the campaign [40]. The presented data will help inform the refinement of targeted melanoma prevention/sun awareness campaigns in Canada that would consider unique weather/geographic variations in this vast multicultural region of the world.

This study had several limitations. The detailed associations do not take into account Fitzpatrick skin phototypes which, as documented in our previous report, vary by province [14] or genetic background of individuals, which may predispose them to CM. Furthermore, the presented model does not account for differences in cultural/social beliefs, attitudes in different regions or socioeconomic status of different communities, which will be addressed in our future studies (through national and regional surveys). It also does not account for travel to warmer/sunnier climates for leisure/personal, work, or study purposes. This work also does not consider regional variations in melanoma awareness or real/perceived barriers to sun protection practices. Nevertheless, the presented study is based on rigorous national unbiased government registries (e.g., CANUE and CCR) presenting clinically relevant conclusions on weather/geographic variations associated with CM incidence in Canada.

## Key Message

This Canadian population-based study presents clinically relevant conclusions on weather/geographic variations associated with melanoma incidence.

## Statement of Ethics

This study received an exemption from the McGill University Research Ethics Board review.

## Conflict of Interest Statement

The authors declare no competing financial interests.

## Funding Sources

This research is funded by a Proof of Concept Intervention Grant in Primary Prevention of Cancer (Action Grant) of the Canadian Cancer Society and the CIHR-Institute for Cancer Research (CCS Grant #707233/CIHR-ICR Grant #478510). This work was further supported by the CIHR Project Scheme Grant #426655 to Dr. Litvinov, CIHR Catalyst Grant #428712 to Drs. Litvinov, Ghazawi, Mukovozov, Mourad, Cyr, Claveau, Gniadecki, Rahme, Sasseville, and Lagacé, Cancer Research Society (CRS)-CIHR Partnership Grant #25343 to Dr. Litvinov, and by the Fonds de la recherche du Québec − Santé to Dr. Litvinov (#34753, #36769 and #296643).

## Author Contributions

M. Berman-Rosa: conceptualization, methodology, data curation, formal analysis, investigation, and visualization. J. Logan: methodology, data curation, formal analysis, investigation, and writing − review and editing. F.M. Ghazawi: data curation, formal analysis, funding acquisition, visualization, and investigation. M. Le: data curation, formal analysis, and investigation. S. Conte: data curation, formal analysis, investigation, and writing − review and editing. E. Netchiporouk: conceptualization, methodology, funding acquisition, data curation, formal analysis, investigation, visualization, and writing–review and editing. I. Mukovozov, J. Cyr, and A. Mourad: investigation, methodology, funding acquisition, and writing − review and editing. W.H. Miller: visualization, methodology, and writing − review and editing. J. Claveau and R. Gniadecki: visualization, methodology, funding acquisition, and writing − review and editing. D. Sasseville, F. Lagacé, and I. V. Litvinov: resources, supervision, funding acquisition, investigation, methodology, project administration, and writing − review and editing. E. Rahme: supervision, investigation, methodology, funding acquisition, project administration, and writing–review and editing.

## Data Availability Statement

All data generated or analyzed during this study are included in this article and its online supplementary material. Further inquiries can be directed to the corresponding author.

## Supplementary Material

Supplementary data

## Figures and Tables

**Table 1:** National differences in environmental conditions that significantly contribute to increased melanoma incidence between high-risk FSAs and low-risk FSAs

	National (excluding Quebec, territories)		
	FSAs with higher than national average (*n* = 329), mean (IQR)	FSAs with lower than or no difference to national average (*n* = 898), mean (IQR)	*t* test* (*p* value)
Average annual temperature, °C	7.75 (2.27)	6.31 (4.50)	<0.05
Mean daily dose of vitD-weighted UV (J/m^2^) for summer months^a^	6,291.31 (640.47)	6,108.09 (763.88)	<0.05
Average NDVI at 1,000 m	0.44 (0.10)	0.41 (0.16)	<0.05

High-risk FSAs were compared to low-risk FSAs via a two-tailed *t* test and significance was determined (alpha = 0.05). IQR, interquartile range; J/m^2^, joules per meter squared; NDVI, normalized difference vegetation index; vitD, vitamin D.

* Wilcoxon Rank Test performed if 1 or both displayed a non-normal distribution.

a Summer months included June, July, and August.

**Table 2:** Regional differences in the average annual number of days with rain, rain events, highest and average temperature, number of heat events, UV radiation, and the NDVI between high-risk FSAs in melanoma incidence high versus low-risk areas

	Maritime^a^ (*n =* 45)	Newfoundland and Labrador (*n* = 33)			
	mean (IQR)	mean (IQR)	*p* value^c^		
A. Comparison of maritime provinces (New Brunswick, Prince Edward Island, Nova Scotia) high-risk region versus Newfoundland and Labrador melanoma low-risk region					
Average annual number of days with rain	106.22 (5.62)	113.25 (13.72)	<0.05		
Average annual number of rain events	48.51 (1.45)	44.19 (3.47)	<0.05		
Average annual highest temperature, °C	30.93 (0.76)	28.06 (1.68)	<0.05		
Average annual number of heat events	1.95 (0.40)	2.28 (0.42)	<0.05		
Average annual temperature, °C	6.91 (0.81)	4.76 (1.01)	<0.05		
Mean daily dose of vitD-weighted UV, * J/m^2^ for summer months^b^	5,813.75 (96.81)	5,051.53 (99.04)	<0.05		
Average NDVI at 1,000 m	0.49 (0.09)	0.47 (0.08)	<0.05		
	Southern Ontario (*n* = 155)	Northern Ontario (*n* = 45)			
	mean (IQR)	mean (IQR)	*p* value^d^		
B. Comparison of Southern Ontario high-risk region versus Northern Ontario melanoma low-risk region					
Average annual number of days with rain	105.12 (6.23)	97.08 (8.72)	<0.05		
Average annual number of rain events	49.93 (4.55)	40.92 (5.42)	<0.05		
Average annual highest temperature, °C	32.63 (1.06)	31.60 (1.47)	<0.05		
Average annual number of heat events	2.4 (0.18)	2.14 (0.35)	<0.05		
Average annual temperature, °C	7.92 (1.42)	3.90 (2.07)	<0.05		
Mean daily dose of vitD-weighted UV, J/m^2^ for summer months^b^	6,530.05 (107.01)	6,077.01 (270.40)	<0.05		
Average NDVI at 1,000 m	0.45 (0.07)	0.47 (0.12)	0.12		
	Southern Ontario (*n* = 155)	Saskatchewan and Manitoba (*n =* 102)		Alberta (*n* = 131)	
	mean (IQR)	mean (IQR)	*p* value^d^	mean (IQR)	*p* value^d^
C. Comparison of Southern Ontario high-risk region versus the adjacent Prairie					
Provinces melanoma low-risk regions					
Average annual number of days with rain	105.12 (6.23)	67.08 (9.21)	<0.05	67.41	<0.05
Average annual number of rain events	49.93 (4.55)	32.95 (4.13)	<0.05	31.06 (2.48)	<0.05
Average annual highest temperature, °C	32.63 (1.06)	33.64 (1.38)	<0.05	32.29 (0.57)	<0.05
Average annual number of heat events	2.4 (0.18)	1.62 (0.22)	<0.05	2.21 (0.22)	<0.05
Average annual temperature, °C	7.92 (1.42)	2.89 (0.44)	<0.05	3.48 (1.18)	<0.05
Mean daily dose of vitD-weighted UV, J/m^2^ for summer months^b^	6,530.05 (107.01)	6,130.43 (210.20)	<0.05	6,007.07	<0.05
				(823.14)	
Average NDVI at 1,000 m	0.45 (0.07)	0.36 (0.10)	<0.05	0.35 (0.07)	<0.05
	Southern British Columbia (*n =* 82)	Saskatchewan and Manitoba (*n =* 102)		Alberta (*n* = 131)	
	mean (IQR)	mean (IQR)	*p* value^e^	mean (IQR)	*p* value^e^
D. Comparison of southern British Columbia high-risk region versus the adjacent Prairie Provinces melanoma low-risk regions.					
Average annual number of days with rain	155.63 (21.02)	67.08 (9.21)	<0.05	67.41	<0.05
Average annual number of rain events	43.74 (1.22)	32.95 (4.13)	<0.05	31.06 (2.48)	<0.05
Average annual highest temperature, °C	32.06 (3.02)	33.64 (1.38)	<0.05	32.29 (0.57)	<0.05
Average annual number of heat events	2.79 (0.28)	1.62 (0.22)	<0.05	2.21 (0.22)	<0.05
Average annual temperature, °C	9.92 (1.29)	2.89 (0.44)	<0.05	3.48 (1.18)	<0.05
Mean daily dose of vitD-weighted UV, J/m^2^ for summer months^b^	6,120.20 (389.37)	6,130.43 (210.20)	<0.05	6,007.07	<0.05
				(823.14)	
Average NDVI at 1,000 m	0.43 (0.09)	0.36 (0.10)	<0.05	0.35 (0.07)	<0.05
	Southern British Columbia (*n =* 82)	Northern British Columbia (*n* = 14)	
	mean (IQR)	mean (IQR)	*p* value^e^		
E. Comparison of southern British Columbia high-risk region versus northern British Columbia melanoma low-risk region					
Average annual number of days with rain	155.63 (21.02)	124.88 (94.81)	<0.05		
Average annual number of rain events	43.74 (1.22)	35.84 (6.30)	<0.05		
Average annual highest temperature, °C	32.06 (3.02)	29.53 (3.40)	0.19		
Average annual number of heat events	2.79 (0.28)	2.37 (0.50)	<0.05		
Average annual temperature, °C	9.92 (1.29)	4.54 (4.01)	<0.05		
Mean daily dose of vitD-weighted UV, J/m^2^ for summer months^b^	6,120.20 (389.37)	5,127.76 (441.83)	<0.05		
Average NDVI at 1,000 m	0.43 (0.09)	0.42 (0.10)	0.83		

Comparisons presented for neighboring jurisdictions. Comparisons were performed via a two-tailed *t* test and significance was determined (alpha = 0.05). BC, British Columbia; IQR, interquartile range; J/m^2^, joules per meter squared; NDVI, normalized difference vegetation index; vitD, vitamin D; FSA, Forward Sortation Area.

* Wilcoxon Rank Test performed if 1 or both displayed a non-normal distribution.

a Maritime Canada includes the provinces of Nova Scotia, New Brunswick, and Prince Edward Island.

b Summer months included June, July, and August.

c *p* value for comparison with region 1 (Maritime Canada) high incidence FSAs.

d *p* value for comparison with region 2 (Southern Ontario) high incidence FSAs.

e *p* value for comparison with region 3 (southern British Columbia) high incidence FSAs.

**Table 3:** Negative binomial generalized linear model parameters, standard error, and significance at *p* < 0.05

Term	Coefficient	IRR	95% CI		Standard error (SE)	*p* value
Intercept	−5.96				0.013	2.00E-16
Average number of annual heat events	−0.036	0.97	0.93	1.00	0.016	0.026
Mean daily dose of vitD-weighted UV J/m^2^ for summer months	0.13	1.14	1.09	1.18	0.020	6.29E-11
Average NDVI at 1,000 m	0.15	1.17	1.13	1.20	0.015	2.00E-16
Average annual number of rain events	−0.12	0.88	0.85	0.92	0.021	4.72E-09
Average highest annual temperature, °C	−0.064	0.94	0.90	0.97	0.018	0.00049
Average annual temperature, °C	0.19	1.21	1.16	1.27	0.024	4.95E-16

AIC: 9,403.2. Stepwise variable selection was performed, and variables selected according to best model fit as assessed by the Akaike Information Criterion (AIC). J/m^2^, joules per meter squared; NDVI, normalized difference vegetation index; vitD, vitamin D. All variables are presented in “*Z*-score” units. Coefficients represent the change in the number of melanoma cases that could be expected in an FSA for a one standard deviation increase above the variable's mean. For instance, (based on IRR value) one might expect a 21.1% increase in the number of cases in an FSA where the annual average temperature exceeded the national mean by one standard deviation. Selection of parameters for the model started with: average annual number of heat events, average annual length of heat events, average annual number of rain events, average annual highest temperature, average annual temperature, mean daily dose of vitD-weighted UV J/m^2^ for summer months, average NDVI at 1,000 m.
