# Supplementary material for: Analysis of Geographic and Environmental Factors and Their Association with Cutaneous Melanoma Incidence in Canada
Source: Dermatology. 2022 Jun 9;238(6):1006–17. doi: 10.1159/000524949 (PMC9677843; doi:10.1159/000524949)
Supplement: Supplementary file 1 — Supplementary data [file drm-0238-1006-s01.pdf]

Table S1. Forward Sortation Areas (FSAs) included within 8 defined geographic regions, categorized as high *vs.* low CM incidence according to their crude incidence rate of CM per 100 000 individuals during 1992-2010 time period.

Region 1 – Maritime Provinces	Region 2 - southern Ontario,	Region 3- British Columbia, excluding northern region.	Region 4- northern Ontario	Region 5- Newfoundland and Labrador	Region 6- Saskatchewan and Manitoba	Region 7- Alberta	Region 8- northern British Columbia
B0A	K0A	V0A	P0A	A0A	R0A	T0A	V0C
B0C	K0B	V0B	P0B	A0B	R0B	T0B	V0J
B0E	K0C	V0E	P0C	A0C	R0C	T0C	V0T
B0H	K0E	V0G	P0E	A0E	R0E	T0E	V0V
B0J	K0G	V0H	P0G	A0G	R0G	T0G	V0W
B0K	K0H	V0K	P0H	A0H	R0H	T0H	V1G
B0L	K0J	V0L	P0J	A0J	R0J	T0J	V1J
B0M	K0K	V0M	P0K	A0K	R0K	T0K	V2K
B0N	K0L	V0N	P0L	A0L	R0L	T0L	V2L
B0P	K0M	V0P	P0M	A0M	R0M	T0M	V2M
B0R	K1A	V0R	P0N	A0N	R1A	T0P	V2N
B0S	K1B	V0S	P0P	A0P	R1B	T0V	V8C
B0T	K1C	V0X	P0R	A0R	R1N	T1A	V8G
B0V	K1E	V1A	P0S	A1A	R2C	T1B	
B0W	K1G	V1B	P0T	A1B	R2E	T1C	
B1A	K1H	V1C	P0V	A1C	R2G	T1G	
B1B	K1J	V1E	P0W	A1E	R2H	T1H	
B1C	K1K	V1H	P0X	A1G	R2J	T1J	
B1E	K1L	V1K	P0Y	A1H	R2K	T1K	
B1G	K1M	V1L	P1A	A1K	R2L	T1L	
B1H	K1N	V1M	P1B	A1L	R2M	T1M	
B1J	K1P	V1N	P1C	A1M	R2N	T1P	

B1K	K1R	V1P	P1H	A1N	R2P	T1R	
B1L	K1S	V1R	P1L	A1S	R2R	T1S	
B1M	K1T	V1S	P1P	A1V	R2V	T1V	
B1N	K1V	V1T	P2A	A1W	R2W	T1W	
B1P	K1W	V1V	P2B	A1X	R2X	T1X	
B1R	K1X	V1W	P2N	A1Y	R2Y	T1Y	
B1S	K1Y	V1X	P3A	A2A	R3A	T1Z	
B1T	K1Z	V1Y	P3B	A2B	R3B	T2A	
B1V	K2A	V1Z	P3C	A2H	R3C	T2B	
B1W	K2B	V2A	P3E	A2N	R3E	T2C	
B1X	K2C	V2B	P3G	A2V	R3G	T2E	
B1Y	K2E	V2C	P3L	A5A	R3H	T2G	
B2A	K2G	V2E	P3N	A8A	R3J	T2H	
B2C	K2H	V2G	P3P		R3K	T2J	
B2E	K2J	V2H	P3Y		R3L	T2K	
B2G	K2K	V2J	P4N		R3M	T2L	
B2H	K2L	V2P	P4P		R3N	T2M	
B2J	K2M	V2R	P4R		R3P	T2N	
B2N	K2P	V2S	P5A		R3R	T2P	
B2R	K2R	V2T	P5E		R3S	T2R	
B2S	K2S	V2V	P5N		R3T	T2S	
B2T	K2T	V2W	P6A		R3V	T2T	
B2V	K2V	V2X	P6B		R3W	T2V	
B2W	K2W	V2Y	P6C		R3X	T2W	
B2X	K4A	V2Z	P7A		R3Y	T2X	
B2Y	K4B	V3A	P7B		R4A	T2Y	
B2Z	K4C	V3B	P7C		R4H	T2Z	
B3A	K4K	V3C	P7E		R4J	T3A	
B3B	K4M	V3E	P7G		R4K	T3B	
B3E	K4P	V3G	P7J		R4L	T3C	

B3G	K4R	V3H	P7K		R5A	T3E	
B3H	K6A	V3J	P7L		R5G	T3G	
B3J	K6H	V3K	P8N		R5H	T3H	
B3K	K6J	V3L	P8T		R6M	T3J	
B3L	K6K	V3M	P9A		R6W	T3K	
B3M	K6T	V3N	P9N		R7A	T3L	
B3N	K6V	V3P			R7B	T3M	
B3P	K7A	V3R			R7C	T3N	
B3R	K7C	V3S			R7N	T3P	
B3S	K7G	V3T			R8A	T3R	
B3T	K7H	V3V			R8N	T3S	
B3V	K7K	V3W			R9A	T3Z	
B3Z	K7L	V3X			S0A	T4A	
B4A	K7M	V3Y			S0C	T4B	
B4B	K7N	V3Z			S0E	T4C	
B4C	K7P	V4A			S0G	T4E	
B4E	K7R	V4B			S0H	T4G	
B4G	K7S	V4C			S0J	T4H	
B4H	K7V	V4E			S0K	T4J	
B4N	K8A	V4G			S0L	T4L	
B4P	K8B	V4K			S0M	T4N	
B4R	K8H	V4L			S0N	T4P	
B4V	K8N	V4M			S0P	T4R	
B5A	K8P	V4N			S2V	T4S	
B6L	K8R	V4P			S3N	T4T	
B9A	K8V	V4R			S4A	T4V	
C0A	K9A	V4S			S4H	T4X	
C0B	K9H	V4T			S4L	T5A	
C1A	K9J	V4V			S4N	T5B	
C1B	K9K	V4W			S4P	T5C	

C1C	K9L	V4X			S4R	T5E	
C1E	K9V	V4Z			S4S	T5G	
C1N	L0A	V5A			S4T	T5H	
E0A	L0B	V5B			S4V	T5J	
E0B	L0C	V5C			S4W	T5K	
E0C	L0E	V5E			S4X	T5L	
E0E	L0G	V5G			S4Y	T5M	
E0G	L0H	V5H			S4Z	T5N	
E0H	L0J	V5J			S6H	T5P	
E0J	L0K	V5K			S6J	T5R	
E0K	L0L	V5L			S6K	T5S	
E0L	L0M	V5M			S6V	T5T	
E1A	L0N	V5N			S6W	T5V	
E1B	L0P	V5P			S6X	T5W	
E1C	L0R	V5R			S7H	T5X	
E1E	L0S	V5S			S7J	T5Y	
E1G	L1A	V5T			S7K	T5Z	
E1H	L1B	V5V			S7L	T6A	
E1J	L1C	V5W			S7M	T6B	
E1N	L1E	V5X			S7N	T6C	
E1V	L1G	V5Y			S7P	T6E	
E1W	L1H	V5Z			S7R	T6G	
E1X	L1J	V6A			S7S	T6H	
E2A	L1K	V6B			S7T	T6J	
E2E	L1L	V6C			S7V	T6K	
E2G	L1M	V6E			S7W	T6L	
E2H	L1N	V6G			S9A	T6M	
E2J	L1P	V6H			S9H	T6N	
E2K	L1R	V6J			S9V	T6P	
E2L	L1S	V6K			S9X	T6R	

E2M	L1T	V6L				T6S	
E2N	L1V	V6M				T6T	
E2P	L1W	V6N				T6V	
E2R	L1X	V6P				T6W	
E2S	L1Y	V6R				T6X	
E2V	L1Z	V6S				T7A	
E3A	L2A	V6T				T7E	
E3B	L2E	V6V				T7N	
E3C	L2G	V6W				T7P	
E3E	L2H	V6X				T7S	
E3G	L2J	V6Y				T7V	
E3L	L2M	V6Z				T7X	
E3N	L2N	V7A				T7Y	
E3V	L2P	V7B				T7Z	
E3Y	L2R	V7C				T8A	
E3Z	L2S	V7E				T8B	
E4A	L2T	V7G				T8C	
E4B	L2V	V7H				T8E	
E4C	L2W	V7J				T8G	
E4E	L3B	V7K				T8H	
E4G	L3C	V7L				T8L	
E4H	L3K	V7M				T8N	
E4J	L3M	V7N				T8R	
E4K	L3P	V7P				T8S	
E4L	L3R	V7R				T8T	
E4M	L3S	V7S				T8V	
E4N	L3T	V7T				T8W	
E4P	L3V	V7V				T8X	
E4R	L3X	V7W				T9A	
E4S	L3Y	V7X				T9C	

E4T	L3Z	V7Y				T9E	
E4V	L4A	V8A				T9G	
E4W	L4B	V8B				T9H	
E4X	L4C	V8J				T9J	
E4Y	L4E	V8K				T9K	
E4Z	L4G	V8L				T9M	
E5A	L4H	V8M				T9N	
E5B	L4J	V8N				T9S	
E5C	L4K	V8P				T9V	
E5E	L4L	V8R				T9W	
E5G	L4M	V8S				T9X	
E5H	L4N	V8T					
E5J	L4P	V8V					
E5K	L4R	V8W					
E5L	L4S	V8X					
E5M	L4T	V8Y					
E5N	L4V	V8Z					
E5P	L4W	V9A					
E5R	L4X	V9B					
E5S	L4Y	V9C					
E5T	L4Z	V9E					
E5V	L5A	V9G					
E6A	L5B	V9H					
E6B	L5C	V9J					
E6C	L5E	V9K					
E6E	L5G	V9L					
E6G	L5H	V7A					
E6H	L5J	V7B					
E6J	L5K	V7C					
E6K	L5L	V7E					

E6L	L5M	V7G					
E7A	L5N	V7H					
E7B	L5P	V7J					
E7C	L5R	V7K					
E7E	L5S	V7L					
E7G	L5T	V7M					
E7H	L5V	V7N					
E7J	L5W	V7P					
E7K	L6A	V7R					
E7L	L6B	V7S					
E7M	L6C	V7T					
E7N	L6E	V7V					
E7P	L6G	V7W					
E8A	L6H	V7X					
E8B	L6J	V7Y					
E8C	L6K	V8A					
E8E	L6L	V8B					
E8G	L6M	V8C					
E8J	L6P	V8G					
E8K	L6R	V8J					
E8L	L6S	V8K					
E8M	L6T	V8L					
E8N	L6V	V8M					
E8P	L6W	V8N					
E8R	L6X	V8P					
E8S	L6Y	V8R					
E8T	L6Z	V8S					
E9A	L7A	V8T					
E9B	L7B	V8V					
E9C	L7C	V8W					

E9E	L7E	V8X					
E9G	L7G	V8Y					
E9H	L7J	V8Z					
	L7K	V9A					
	L7L	V9B					
	L7M	V9C					
	L7N	V9E					
	L7P	V9G					
	L7R	V9H					
	L7S	V9J					
	L7T	V9K					
	L7V	V9L					
	L8E	V9M					
	L8G	V9N					
	L8H	V9P					
	L8J	V9R					
	L8K	V9S					
	L8L	V9T					
	L8M	V9V					
	L8N	V9W					
	L8P	V9X					
	L8R	V9Y					
	L8S	V9Z					
	L8T						
	L8V						
	L8W						
	L9A						
	L9B						
	L9C						
	L9G						

	L9H						
	L9J						
	L9K						
	L9L						
	L9M						
	L9N						
	L9P						
	L9R						
	L9S						
	L9T						
	L9V						
	L9W						
	L9X						
	L9Y						
	L9Z						
	M1B						
	M1C						
	M1E						
	M1G						
	M1H						
	M1J						
	M1K						
	M1L						
	M1M						
	M1N						
	M1P						
	M1R						
	M1S						
	M1T						
	M1V						

	M1W						
	M1X						
	M2H						
	M2J						
	M2K						
	M2L						
	M2M						
	M2N						
	M2P						
	M2R						
	M3A						
	M3B						
	M3C						
	M3H						
	M3J						
	M3K						
	M3L						
	M3M						
	M3N						
	M4A						
	M4B						
	M4C						
	M4E						
	M4G						
	M4H						
	M4J						
	M4K						
	M4L						
	M4M						
	M4N						

	M4P						
	M4R						
	M4S						
	M4T						
	M4V						
	M4W						
	M4X						
	M4Y						
	M5A						
	M5B						
	M5C						
	M5E						
	M5G						
	M5H						
	M5J						
	M5K						
	M5L						
	M5M						
	M5N						
	M5P						
	M5R						
	M5S						
	M5T						
	M5V						
	M5W						
	M5X						
	M6A						
	M6B						
	M6C						
	M6E						

	M6G						
	M6H						
	M6J						
	M6K						
	M6L						
	M6M						
	M6N						
	M6P						
	M6R						
	M6S						
	M7A						
	M7R						
	M7X						
	M7Y						
	M7Z						
	M8V						
	M8W						
	M8X						
	M8Y						
	M8Z						
	M9A						
	M9B						
	M9C						
	M9L						
	M9M						
	M9N						
	M9P						
	M9R						
	M9V						
	M9W						

	N0A						
	N0B						
	N0C						
	N0E						
	N0G						
	N0H						
	N0J						
	N0K						
	N0L						
	N0M						
	N0N						
	N0P						
	N0R						
	N1A						
	N1C						
	N1E						
	N1G						
	N1H						
	N1K						
	N1L						
	N1M						
	N1P						
	N1R						
	N1S						
	N1T						
	N2A						
	N2B						
	N2C						
	N2E						
	N2G						

	N2H						
	N2J						
	N2K						
	N2L						
	N2M						
	N2N						
	N2P						
	N2R						
	N2S						
	N2T						
	N2V						
	N2Z						
	N3A						
	N3B						
	N3C						
	N3E						
	N3H						
	N3L						
	N3P						
	N3R						
	N3S						
	N3T						
	N3V						
	N3W						
	N3Y						
	N4B						
	N4G						
	N4K						
	N4L						
	N4N						

	N4S						
	N4T						
	N4V						
	N4W						
	N4X						
	N4Z						
	N5A						
	N5C						
	N5H						
	N5L						
	N5P						
	N5R						
	N5V						
	N5W						
	N5X						
	N5Y						
	N5Z						
	N6A						
	N6B						
	N6C						
	N6E						
	N6G						
	N6H						
	N6J						
	N6K						
	N6L						
	N6M						
	N6N						
	N6P						
	N7A						

	N7G						
	N7L						
	N7M						
	N7S						
	N7T						
	N7V						
	N7W						
	N7X						
	N8A						
	N8H						
	N8M						
	N8N						
	N8P						
	N8R						
	N8S						
	N8T						
	N8V						
	N8W						
	N8X						
	N8Y						
	N9A						
	N9B						
	N9C						
	N9E						
	N9G						
	N9H						
	N9J						
	N9K						
	N9V						
